# Supplementary material for: Comprehensive Annotation and Functional Exploration of MicroRNAs in Lettuce
Source: Front Plant Sci. 2021 Dec 24;12:781836. doi: 10.3389/fpls.2021.781836 (PMC8739914; doi:10.3389/fpls.2021.781836)
Supplement: Supplementary file 2 [file Data_Sheet_8.ZIP › GSM2230747.plot/Lsa-miR171d_Lsat_1_v5_gn_7_9261.1_1164_TPlot.pdf]

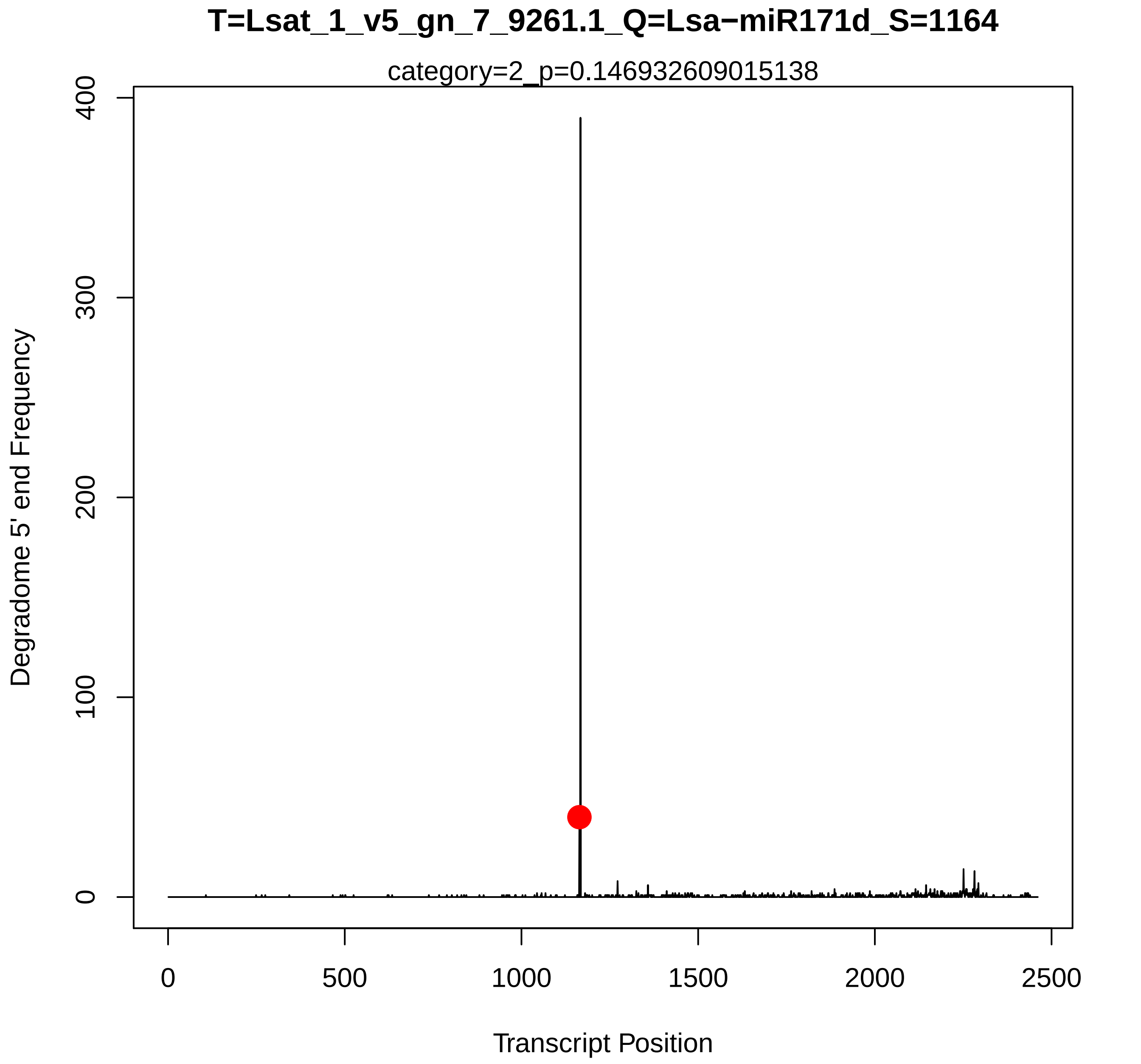

T=Lsat_1_v5_gn_7_9261.1_Q=Lsa−miR171d_S=1164
category=2_p=0.146932609015138
Degradome 5' end Frequency
0
100
200
300
400
0
500
1000
1500
2000
2500
Transcript Position
